# Poly(ethylene oxide)- and Polyzwitterion-Based Thermoplastic Elastomers for Solid Electrolytes

**DOI:** 10.3390/ma17092145

**Published:** 2024-05-03

**Authors:** Ding-Li Xia, Shi-Peng Ding, Ze Ye, Chen Yang, Jun-Ting Xu

**Affiliations:** National Key Laboratory of Biobased Transportation Fuel Technology, International Research Center for X Polymers, Department of Polymer Science and Engineering, Zhejiang University, Hangzhou 310058, China; 3160102760@zju.edu.cn (D.-L.X.); 3150102770@zju.edu.cn (S.-P.D.); 22029025@zju.edu.cn (Z.Y.); 11929024@zju.edu.cn (C.Y.)

**Keywords:** block copolymer, solid electrolytes, polyzwitterion, mechanical strength, electrical performance

## Abstract

In this article, ABA triblock copolymer (tri-BCP) thermoplastic elastomers with poly(ethylene oxide) (PEO) middle block and polyzwitterionic poly(4-vinylpyridine) propane-1-sulfonate (PVPS) outer blocks were synthesized. The PVPS*-b-*PEO*-b-*PVPS tri-BCPs were doped with lithium bis-(trifluoromethane-sulfonyl) imide (LiTFSI) and used as solid polyelectrolytes (SPEs). The thermal properties and microphase separation behavior of the tri-BCP/LiTFSI hybrids were studied. Small-angle X-ray scattering (SAXS) results revealed that all tri-BCPs formed asymmetric lamellar structures in the range of PVPS volume fractions from 12.9% to 26.1%. The microphase separation strength was enhanced with increasing the PVPS fraction (*f*_PVPS_) but was weakened as the doping ratio increased, which affected the thermal properties of the hybrids, such as melting temperature and glass transition temperature, to some extent. As compared with the PEO/LiTFSI hybrids, the PVPS*-b-*PEO*-b-*PVPS/LiTFSI hybrids could achieve both higher modulus and higher ionic conductivity, which were attributed to the physical crosslinking and the assistance in dissociation of Li^+^ ions by the PVPS blocks, respectively. On the basis of excellent electrical and mechanical performances, the PVPS*-b-*PEO*-b-*PVPS/LiTFSI hybrids can potentially be used as solid electrolytes in lithium-ion batteries.

## 1. Introduction

As lithium-ion batteries (LIBs) possess the advantages of high energy density, long lifespan, and high charging speed, they are becoming increasingly prevalent in recent years [1,2,3]. Despite the increase in efficiency and convenience generated by LIBs, commercially used liquid electrolytes in LIBs may lead to some potential problems, such as dendrite formation, fire, and explosion [4,5,6]. Utilizing solid polymer electrolytes (SPEs) in place of liquid electrolytes could be a prospective solution. SPEs have been the focal point of research advancements since their capability to dissolve lithium salts effectively was pioneered by Wright et al. [7], with a significant body of subsequent studies dedicated to the optimization of SPEs’ architecture [8,9,10,11]. SPEs with high shear moduli possess excellent processability and are able to inhibit dendrite growth [12,13,14]. The predominant challenges associated with SPEs revolve around harmonizing ionic conductivity with mechanical strength and enhancing the suboptimal interfacial adherence between the electrode and electrolyte [15,16,17,18]. Innovations such as the integration of novel lithium salts and anions, the copolymerization process, graft-modified polymers, and the formation of composites with inorganic ceramic fillers have shown promise in mitigating these issues [19].

With its excellent ability to dissolve lithium salts effectively and make mobility requisite for Li^+^ ion transport, salt-doped poly(ethylene oxide) (PEO) serves as the most representative polymer electrolyte [20,21]. There are several methods to modify homogeneous PEO materials for higher ionic conductivity and shear modulus, such as regulation of molecular weight [22], salt doping ratio [23], and temperature [24], but the trade-off between polymer chain mobility and stiffness makes it difficult to attain both excellent conductivity and mechanical strength [13,15]. 

Recently, block copolymers (BCPs) have been extensively studied due to their unique self-assembly structures at the nanoscale, which conduces to a combination of various distinctive properties [25,26]. The connection of PEO with a reinforcing block can provide SPE materials with both ion transport channels and improved mechanical strength [27,28]. Hillmyer’s group reported a kind of BCP electrolyte material based on polymerization-induced phase separation and found that the ionic conductivity of the material could reach 10^−3^ S/cm at room temperature with its elastic modulus close to 1 GPa [16]. However, among the majority of BCP materials studied, how to decouple conductivity with modulus completely still remains a problem [13,29], since introducing insulated blocks with better mechanical properties into the electrolytes usually has an adverse impact on ionic conductivity. For example, Balsara et al. showed that lithium bis-(trifluoromethane-sulfonyl) imide (LiTFSI)-doped poly(styrene)*-b-*PEO (PS*-b-*PEO) brought out enhanced modulus but restricted conductivity compared to PEO/LiTFSI [9,30,31]. Notably, our group found that BCPs with double conductive phases that both constructing blocks are able to dissolve Li^+^ ions as SPEs, such as poly(propylene monothiocarbonate)*-b-*PEO (PPMTC*-b-*PEO), could achieve synchronous enhancement of conductivity and modulus via regulating the microphase separation behavior of the double conductive phases [32,33]. In our previous work, we systematically studied the effects of phase structure, grain size, and interphase on the conductivity of block copolymer electrolytes with double-conductive phases [32,33,34,35].

In the past few years, zwitterions and polyzwitterions have been extensively applied for gel polymer electrolytes (GPEs) or SPEs in LIBs [36,37,38,39]. Zwitterions, such as sulfobetaine and carboxybetaine, contain covalently bonded cation and anion in a single molecule, while polyzwitterions represent polymers with repeating units bearing zwitterions [40,41]. Ionic conductivity studies on polyzwitterion/salt systems were carried out by Manero et al., and they found that the conductivity of the systems followed the Arrhenius equation [42]. Segalman and coworkers proposed semicrystalline polymeric zwitterionic (PZI) electrolytes with superionic lithium transport [43]. Unlike the classical vehicular conduction mechanism in polyether and polymeric ionic liquid (PIL) electrolytes [44,45], lithium ion transport in these PZI electrolytes existed in two distinctive circumstances: The amorphous phase led to vehicular motion like traditional SPEs, and the ordered crystalline structure of pendant ZI groups enabled superionic transport similar to inorganic solid-state electrolytes, where Li^+^ ions diffused nearly one order of magnitude more quickly than vehicular motion. Decoupling of ion transport with polymer segmental arrangement and size-selective ion motion for Li^+^ ions facilitated by ordered lattices in the system resulted in high ionic conductivity and lithium transfer number. Macfarlane et al. introduced zwitterion in place of ionic liquid as a plasticizer into polymer electrolytes [46], and the conductivity and diffusion of lithium ions were significantly improved. They postulated that the oleophobic nature of zwitterion could enhance the mobility of Li^+^ ions by providing a polar medium. Yoshizawa-Fujita designed a series of oligoether/zwitterion diblock copolymers and found the ionic conductivity of the SPEs to be maintained even at a high salt doping ratio [47]. They proposed that the aggregation of dissociated ions was hindered by the polar zwitterion structure. Further implementation of these SPEs in cathode coatings for LIBs showed that these copolymers exhibited a substantial electrochemical stabilization window and preserved stable discharge capacities over successive cycles.

According to the strong intramolecular and intermolecular interactions of zwitterion in bulk, the zwitterion phase in zwitterion-containing copolymers presents enormous immiscibility with the other non-charged components [48,49]. The bulk solidity below the glass transition temperature (*T*_g_) makes it possible that the polyzwitterion phase serves as physical cross-linking points, thus enhancing the mechanical strength of the materials [50]. Beyer and Long compared the storage moduli of *n*-butyl acrylate-based zwitterionomers and ionomers [51]. They observed well-defined microphase separation and a rubbery plateau in zwitterionomers only when zwitterion content reached a certain amount.

In this work, ABA triblock copolymers (tri-BCPs) composed of poly(4-vinylpyridine) propane-1-sulfonate (PVPS) (A-block) and PEO (B-block) were used as SPE for LIBs for the purpose of simultaneous improvement in conductivity and mechanical performance. In addition to providing stiffness, the PVPS blocks can also dissociate and dissolve lithium salt, thus enabling the formation of double conductive phases in this type of ABA tri-BCP. The ionic conductivity and mechanical strength of the block copolymer solid electrolytes (BCPSEs), PVPS*-b-*PEO*-b-*PVPS/LiTFSI, with different compositions and doping ratios were investigated. It was observed that some of the electrolytes attained ionic conductivities and storage moduli higher than the PEO/LiTFSI blends. These electrically and mechanically enhanced SPEs are expected to inform the design of PEO-based electrolytes in LIBs in the future.

## 2. Materials and Methods

### 2.1. Materials

Poly(ethylene oxide) (*M*_n_ =10,000 g/mol, PEO_210_), oxalyl chloride (C_2_O_2_Cl_2_, 98%), 2-(dodecylthiocarbonothioylthio)-2-methylpropanoic acid (TTCA, 97%, HPLC), superdry *N*, *N*-dimethylformamide (DMF), azobis(isobutyronitrile) (AIBN), 1,3-propanesultone, and bis(trifluoromethane) sulfonimide lithium (LiTFSI, 98%) were purchased from J&K Scientific Corporation (Beijing, China). Dichloromethane (CH_2_Cl_2_) was bought from TCI (Tokyo, Japan). 4-Vinylpyridine (4-VP), containing stabilizer hydroquinone, was obtained from Sigma-Aldrich (St. Louis, MO, USA). Trifluoroethanol (TFEA) was purchased from Macklin (Shanghai, China). PEO was azeotropically distilled with dry toluene before use. The 4-VP monomer was purified by a neutral alumina column and stored at −10 °C. AIBN was recrystallized from ethanol three times.

### 2.2. Synthesis of PVPS-b-PEO-b-PVPS tri-BCPs

**Scheme 1:**
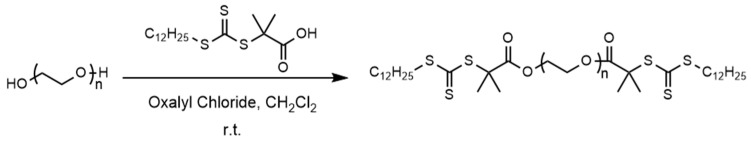

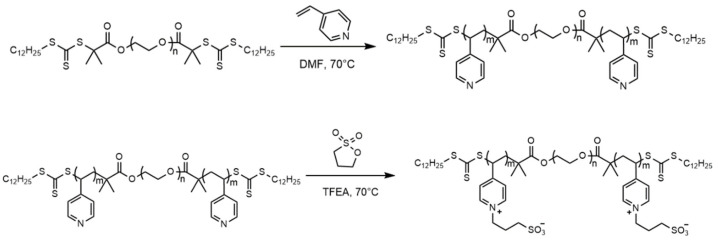
Synthesis route of PVPS*-b-*PEO*-b-*PVPS tri-BCPs.

PVPS*-b-*PEO*-b-*PVPS tri-BCPs were synthesized by reversible addition-fragmentation chain transfer (RAFT) polymerization. First, esterification of PEO with TTCA was carried out in the presence of oxalyl chloride dissolved in CH_2_Cl_2_, and the CTA-PEO-CTA double-ended macromolecular chain transfer agent was prepared. Next, a series of poly(4-vinylpyridine)*-b-*poly(ethylene oxide)*-b-*poly(4-vinylpyridine) (P4VP*-b-*PEO*-b-*P4VP) ABA triblock BCPs were synthesized via RAFT polymerization of 4-VP in DMF. 1,3-propanesultone was then added for further modification of the pyridine group in TFEA. The detailed synthesis process and characterization results of the polymers are provided in the Supplementary Materials. Scheme 1 shows the synthesis route of PVPS*-b-*PEO*-b-*PVPS tri-BCPs. Table 1 lists the molecular characteristics and volume fractions of the PVPS block in tri-BCPs. The detailed volume fractions of PVPS in doped tri-BCPs are given in Table S1. 

### 2.3. Preparation of Polymer/LiTFSI Hybrids

A certain amount of PVPS*_n_-b-*PEO_210_*-b-*PVPS*_n_* (or PEO_210_) and LiTFSI were dissolved in TFEA and stirred for 12 h to obtain homogeneous solutions. After being dried under dynamic vacuum at room temperature for 24 h, the above blends were placed in a vacuum oven for 48 h at 60 °C. Finally, the products were collected and stored in the glove box in an atmosphere of nitrogen. Doping ratio (*r*) is defined as the molar ratio of Li^+^ ions to the sum of EO units and sulfonic acid units, both of which have the ability to complex with Li^+^ ions, i.e., *r* = [Li^+^]/([EO] + [VPS]).

### 2.4. Characterizations

The composition of P4VP-*b*-PEO-*b*-P4VP tri-BCPs was determined by ^1^H-NMR with Bruker DMX 400 (400 MHz) (Bruker Corporation, Billerica, MA, USA) utilizing deuterated chloroform (CDCl_3_) as solvent. Relative molecular weight and polydispersity of PEO and P4VP*-b-*PEO*-b-*P4VP were measured by gel permeation chromatography (GPC) (Waters Corporation, Milford, MA, USA) on a Waters system with DMF as eluent and polystyrene (PS) as standards for calibration. The flow rate of DMF was kept at 1 mL/min while the test temperature was set at 40 °C. Fourier-transform infrared (FTIR) spectra were collected from a Nicolet 6700 spectrometer (Thermo Fisher Scientific Inc., Waltham, MA, USA) with a resolution of 1 cm^−1^. Differential scanning calorimetry (DSC) was carried out on a TA DSC25 (TA Instruments, Wakefield, MA, USA) instrument to characterize the crystallization behavior and glass transition temperature (*T*_g_) of the samples. The tests were performed with heating and cooling rates of 10 °C/min under nitrogen. Temperature-variable small-angle X-ray scattering (SAXS) experiments were operated at beamline BL16B1 (Shanghai Synchrotron Radiation Facility (SSRF), Shanghai, China). Two-dimensional SAXS patterns (2D-SAXS) were recorded by a Pilatus 2M detector (Dectris Ltd., Baden, Switzerland) and transformed into one-dimensional profiles with FIT2D software (https://www.esrf.fr/computing/scientific/FIT2D/ accessed on 20 March 2024). Samples were annealed for 24 h at 120 °C and stepwise cooled to room temperature prior to SAXS testing. The wavelength of the X-ray was 1.24 Å, and the distance between the sample and the detector was 1920 mm. The average exposure time of the samples was 20 s. The scattering vector of the SAXS curves was calibrated with silver behenate. Temperature-variable rheological measurements were performed on a HAAKE RS 6000 rheometer (Thermo Fisher Scientific Inc., MA, USA) using 20 mm diameter parallel plates with a 1 mm gap between the plates. Temperature sweeps with a frequency of 1 Hz between 30 and 100 °C were collected within the linear viscoelastic regime at a heating rate of 2 °C/min under nitrogen. Before the rheological tests, the samples were thermally pressed into films with a thickness of 1 mm and cooled down to room temperature under relatively high pressure. The impedance spectroscopy of the polymer electrolyte films was recorded by an electrochemical workstation (CHI660E, CH Instruments, Inc., Bee Cave, TX, USA) under nitrogen with a frequency range of 1 MHz to 1 Hz and a fixed amplitude of 0.05 V. After being annealed at 120 °C for 24 h and cooled to room temperature, the electrolyte films were sandwiched between two stainless steel electrodes and a Teflon washer. The resistance that originated from ion transport in the polymer electrolytes was determined by the local minimum in the Nyquist plot of the impedance. The conductivities (*σ*) of the electrolytes were calculated by the equation *σ* = *L*/(*R*∙*S*), where *L*, *R,* and *S* represent the electrolyte thickness, resistance, and area, respectively.

## 3. Results and Discussion

### 3.1. Microphase Separation Behavior

**Figure 1:**
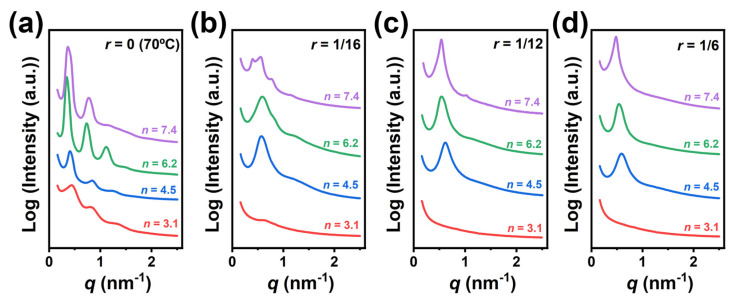
SAXS profiles of PVPS*_n_*−*b*−PEO_210_−*b*−PVPS*_n_*/LiTFSI blends with (**a**) *r* = 0, obtained at 70 °C, (**b**) *r* = 1/16, (**c**) *r* = 1/12, and (**d**) *r* = 1/6 at 30 °C.

Figure 1 shows the SAXS profiles of PVPS*_n_-b-*PEO_210_*-b-*PVPS*_n_* tri-BCPs and PVPS*_n_-b-*PEO_210_*-b-*PVPS*_n_*/LiTFSI hybrids. It is found that, above the melting temperature (*T*_m_) of PEO, all the neat tri-BCPs exhibit ordered lamellar structure as indicated by the relative position of higher-order peaks (*q*) and the primary peak (q*) (*q*: q* = 1: 2: 3:…) in SAXS profiles (Figure 1a). Notably, for traditional non-charged diblock copolymers, only when the volume fraction of a phase reaches 40% to 60% can lamellar structure be formed [53], while in our samples, the volume fractions of PVPS in lamella-forming samples are far below 40%. This is because introducing Coulombic interaction into BCPs may shift the symmetry of the phase diagram, and the lamellar structure formed at a low content of charged phase is a consequence of physical crosslinking among the polyzwitterion blocks [48,49]. With the increase in zwitterionic PVPS content, most samples present a reduced peak width and more high-order peaks, indicating stronger phase separation.

In contrast, as the doping ratio rises, the ordering degree of the microphase-separated structures becomes lower. Hybrids at different doping ratios show ill-defined structures at room temperature, as their SAXS profiles (Figure 1b–d) show no higher-order peaks or higher-order peaks with uncharacteristic proportional position relations to the primary peaks. Homogeneous structures are even formed in PVPS_3.1_*-b-*PEO_210_*-b-*PVPS_3.1_/LiTFSI hybrids at doping ratios of 1/12 and 1/6, as indicated by their flat SAXS profiles (Figure 1c,d). This result is related to the competitive distribution of lithium salt in different blocks, as proved by the FTIR spectra shown in Figure S4. Lithium salt is first distributed in the PEO phase, leading to the charged characteristic of the PEO block, improved similarity with the PVPS block, and decreased microphase separation strength. The shoulder peaks superposed on the primary scattering peaks observed at the doping ratio of 1/16 are attributed to PEO crystals, which will disappear after the fusion of PEO crystals (Figure S5).

Temperature-variable SAXS experiments were carried out for PVPS*_n_-b-*PEO_210_*-b-*PVPS*_n_*/LiTFSI hybrids at *r* = 1/16, among which *n* corresponds to 4.5, 6.2, and 7.4, respectively. In Figure S5, it is observed that the scattering vector ratio of the primary and higher-order peaks remains 1:2 at the temperatures above the melting of PEO crystal, suggesting that these hybrids maintain a lamellar structure.

The effective Flory Huggins parameter (*χ*_eff_) between PEO and PVPS at the doping ratio 1/16 was obtained by fitting the disordered scattering peak of PVPS_3.1_-*b*-PEO_210_-*b*-PVPS_3.1_/LiTFSI hybrids in terms of the theory developed by Leibler [54,55,56]. It is found that *χ*_eff_ of this hybrid is 0.51 at 30 °C (Figure S6), much larger than 0.09 reported for PS*-b-*PEO/LiTFSI hybrids at the same doping ratio and temperature (detailed formula and calculation are given in Supplementary Materials) [30] and 0.092 for PEO_114_*-b-*P4VP_27_ BCP at 60 °C [57]. The strong incompatibility between PEO and PVPS accounts for the maintenance of the ordered structure, which ensures the formation of physical crosslinking points in the thermoplastic elastomers.

### 3.2. Thermal Properties 

**Figure 2:**
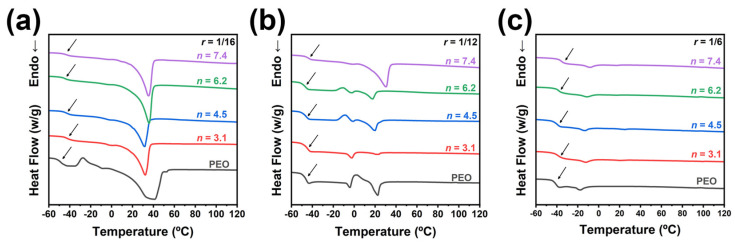
DSC 2nd heating curves of PEO_210_/LiTFSI and PVPS*_n_*−*b*−PEO_210_−*b*−PVPS*_n_*/LiTFSI with different doping ratios. (**a**) *r* = 1/16, (**b**) *r* = 1/12, and (**c**) *r* = 1/6.

The thermal behaviors of PVPS*_n_-b-*PEO_210_*-b-*PVPS*_n_*/LiTFSI hybrids were characterized by DSC (detailed information is given in Table S2). As illustrated in Figure 2, a glass transition at low temperature and an endothermic peak are observed for each sample, while a cold crystallization process was seen in some specific samples. The endothermic peaks are ascribed to the melting of PEO. The crystallinity of PEO given in Table S2 was calculated based on the melting enthalpy, and it was found to decrease with the doping ratio. Notably, when the doping ratio is raised to 1/6, the crystallinity of PEO decreases to below 5%. As a result, the impact of crystallization on the subsequent experiments operated above r.t. for the samples with *r* = 1/6 can be ignored.

It is reported in the literature that the *T*_g_s of PEO and PVPS are −65 °C and 226 °C [58,59], respectively. The *T*_g_s observed in Figure 2 are close to those of PEO, so they are assigned to the PEO-rich phase in the PVPS*_n_-b-*PEO_210_*-b-*PVPS*_n_*/LiTFSI hybrids. After introducing PVPS block into the system, the *T*_g_s of the PEO-rich phase are increased in most cases compared with pure PEO homopolymers. It is found that, with increasing the volume fraction of PVPS, the *T*_g_ becomes lower at first and then increases. Two aspects account for such a trend. When the content of PVPS is lower, the phase separation between PEO and PVPS is incomplete, along with a weaker spatial confinement effect on PEO segments, but there are more rigid PVPS segments in the PEO-rich phase. On the other hand, the degree of microphase separation is high at a larger volume fraction of PVPS, resulting in a lower PVPS content in the PEO-rich phase but a stronger spatial restriction on the segmental motion of PEO. As the doping ratio increases from 1/16 to 1/6, *T*_g_ of the PEO-rich phase in PVPS_7.4_-*b*-PEO_210_-*b*-PVPS_7.4_/LiTFSI hybrids increases on account of suppression of PEO segmental motion by disassociated LiTFSI salt. However, *T*_g_s of PVPS_4.5_*-b-*PEO_210_*-b-*PVPS_4.5_/LiTFSI and PVPS_6.2_*-b-*PEO_210_*-b-*PVPS_6.2_/LiTFSI hybrids first decrease then increase with increasing salt content, while it takes a monotonous uptrend in PEO/LiTFSI hybrids. The phenomenon observed in the above tri-BCP/LiTFSI hybrids is ascribed to the enhancement in dissociation of LiTFSI by introducing polyzwitterion blocks, and such a downward trend in *T*_g_s has also been reported in oligoether/zwitterion diblock copolymers doped with LiTFSI [47].

Changes in crystallization and glass transition behaviors have a significant impact on the segmental motion of PEO chains. Lower crystallinity and *T*_g_ of PEO correspond to better mobility and less stiffness of the polymer chain, which is beneficial for the increase in conductivity [20]. 

### 3.3. Rheological Properties

**Figure 3:**
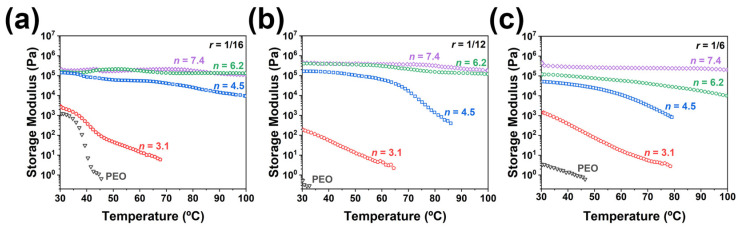
Storage moduli of PVPS*_n_*−*b*−PEO_210_−*b*−PVPS*_n_*/LiTFSI and PEO_210_/LiTFSI hybrids at different doping ratios. (**a**) *r* =1/16, (**b**) *r* = 1/12, and (**c**) *r* = 1/6.

Figure 3 shows the variation of storage modulus (*G*′) with temperature for the PVPS*_n_-b-*PEO_210_*-b-*PVPS*_n_*/LiTFSI and PEO_210_/LiTFSI hybrids at different doping ratios. The initial *G*′ of PEO_210_/LiTFSI hybrids at 30 °C ranges from 10^0^ to 10^3^ Pa and decreases with increasing temperature. As the doping ratio increases, the initial storage modulus of PEO/LiTFSI hybrids drops at first and then becomes a little larger. This originates from two opposite aspects. On the one hand, salt doping restricts the crystallizability of PEO, thus impairing its mechanical properties. On the other hand, salt also serves as a crosslinking point for the homogeneous melt of PEO, which will enhance the modulus.

All the PVPS*_n_-b-*PEO_210_*-b-*PVPS*_n_*/LiTFSI hybrids exhibit higher *G*′ values than the PEO_210_/LiTFSI hybrids due to the reinforcement of the PVPS phase. Hybrids with a larger fraction of the PVPS block exhibit a higher *G*′ at the same doping ratio. The value of *G*′ for PVPS_7.4_*-b-*PEO_210_*-b-*PVPS_7.4_/LiTFSI hybrid at *r* = 1/6 can reach 3 × 10^5^ Pa, which is at least 10^4^ times higher than that of PEO_210_/LiTFSI hybrid at the same doping ratio. It is also observed that, with increasing temperature, the modulus of some tri-BCP hybrids first maintains a plateau in the low temperature range and then decreases gradually at higher temperatures. The turning point shifts to a higher temperature as *f*_PVPS_ in the tri-BCPs increases. Since the temperature corresponding to the turning point can be higher than the *T*_m_ of PEO and no order-to-disorder transition is observed around the turning point (Figure S7), the decrease of *G*′ may be attributed to the pulling-out of partial PVPS blocks from the physical crosslinking domains under dynamic shearing. The physical crosslinking domains become more stable at a higher *f*_PVPS_, and as a consequence, the PVPS_7.4_*-b-*PEO_210_*-b-*PVPS_7.4_/LiTFSI hybrids even keep nearly constant moduli in the temperature range studied (30–100 °C). The relatively high and constant moduli of the PVPS_7.4_*-b-*PEO_210_*-b-*PVPS_7.4_/LiTFSI hybrids in a broad temperature range are advantageous to the processing and application of SPEs for LIBs with good safety. It is reported that lithium dendrites can be suppressed when the shear modulus of the separator (approximately 6 GPa) is 1.8 times higher than that of Li metal (4.9 GPa) [60]. Although the overall moduli of the PVPS*-b-*PEO*-b-*PVPS/LiTFSI hybrids are at the magnitude of 10^5^ Pa, due to the microphase-separated structure formed in the hybrids, the growth of lithium dendrites cannot bypass the PVPS microdomains, and it will be stopped when meeting the glassy PVPS microdomains with a high modulus.

### 3.4. Ionic Conductivity

**Figure 4:**
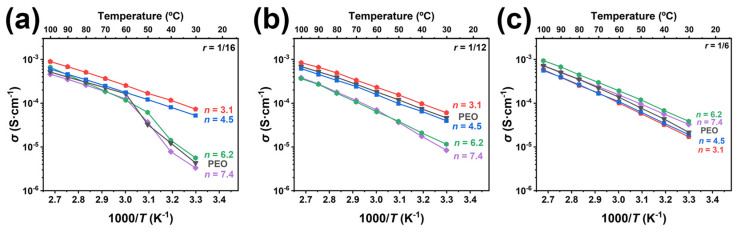
Ionic conductivities (*σ*) of PVPS*_n_*−*b*−PEO_210_−*b*−PVPS*_n_*/LiTFSI and PEO_210_/LiTFSI hybrids at different doping ratios. (**a**) *r* =1/16, (**b**) *r* = 1/12, and (**c**) *r* = 1/6.

The electrical performances of PVPS*_n_-b-*PEO_210_*-b-*PVPS*_n_*/LiTFSI hybrids were characterized by the impedance spectrum. Figure 4 illustrates the ionic conductivity (*σ*) of PVPS*_n_-b-*PEO_210_*-b-*PVPS*_n_*/LiTFSI and PEO_210_/LiTFSI hybrids at different doping ratios. It is found that, at *r* = 1/16 and 1/12, the ionic conductivities of PVPS_3.1_*-b-*PEO_210_*-b-*PVPS_3.1_/LiTFSI are higher than those of PEO_210_/LiTFSI, and PVPS_4.5_*-b-*PEO_210_*-b-*PVPS_4.5_/LiTFSI exhibits ionic conductivities comparable to PEO_210_/LiTFSI. This may be due to the relative enrichment of LiTFSI in the PEO phase, which is frequently observed in the hybrids of salt and BCPs where both blocks can complex with salt, especially at low doping ratios [33,34,61]. With further increasing the PVPS fraction in the tri-BCPs, the ionic conductivities of PVPS_6.2_*-b-*PEO_210_*-b-*PVPS_6.2_/LiTFSI and PVPS_7.4_*-b-*PEO_210_*-b-*PVPS_7.4_/LiTFSI become lower than those of PEO_210_/LiTFSI at the same doping ratio. Notably, Table S2 shows that the minimal *T*_g_ of PEO occurs in the PVPS_6.2_*-b-*PEO_210_*-b-*PVPS_6.2_/LiTFSI hybrids, suggesting a lower concentration of PVPS in the PEO-rich phase due to enhanced microphase separation. This implies that at this juncture, the limitation of chain segment motion is not the predominant influence on conductivity. Furthermore, the grain sizes resulting from microphase separation for each sample set, as detailed in Table S3, do not adequately justify the reduced conductivity observed at elevated PVPS content with low salt-doping ratios [62,63]. It is postulated that with increased PVPS content, microphase separation becomes more pronounced and the phase interfaces are more defined. Consequently, at lower lithium salt concentrations, salts predominantly localize within the interstitial zones of the PEO phase, akin to the PS-*b*-PEO system [25]. This distribution limits the efficacy of PVPS in facilitating salt dissociation, and the morphology effect and grain boundary effect culminate in actual conductivities that are less than theoretical predictions. In the hybrids of tri-BCP, the nominal doping ratio is denoted as *r* = [LiTFSI]/([EO] + [VPS]). Since the PEO block has stronger association ability toward Li^+^ ions than the PVPS block, the salt concentration in the PEO phase of tri-BCPs hybrids is higher than that in PEO_210_/LiTFSI, leading to the higher conductivity of the former. We also notice that the ionic conductivities of PVPS_6.2_*-b-*PEO_210_*-b-*PVPS_6.2_/LiTFSI and PVPS_7.4_*-b-*PEO_210_*-b-*PVPS_7.4_/LiTFSI at 1/16 are quite low in the low temperature range. This can be attributed to the presence of un-melted PEO crystals at testing temperatures, as confirmed by their higher *T*_m_s of PEO (Figure 2 and Table S2). 

At *r* =1/6, the situation becomes reversed. The ionic conductivities of PVPS_6.2_*-b-*PEO_210_*-b-*PVPS_6.2_/LiTFSI and PVPS_7.4_*-b-*PEO_210_*-b-*PVPS_7.4_/LiTFSI hybrids are higher than those of PEO_210_/LiTFSI, whereas the PVPS_3.1_*-b-*PEO_210_*-b-*PVPS_3.1_/LiTFSI and PVPS_4.5_*-b-*PEO_210_*-b-*PVPS_4.5_/LiTFSI hybrids exhibit lower ionic conductivities than PEO_210_/LiTFSI. This can be attributed to the unique dipole nature of zwitterionic molecules, which probably induce the Li^+^ ions to dissociate from the lithium salt. In the literature, it was also reported that the introduction of zwitterion or polyzwitterion into the electrolytes by blending [64] or random copolymerization [65] could improve the electrical performance. It should be noted that the effect of helping dissociate Li^+^ ions becomes obvious only at high doping ratios and in tri-BCPs with high PVPS fractions, since at low doping ratios, there are few salts in the PVPS phase with weaker association ability. This also leads to the high ionic conductivities of PVPS_6.2_*-b-*PEO_210_*-b-*PVPS_6.2_/LiTFSI and PVPS_7.4_*-b-*PEO_210_*-b-*PVPS_7.4_/LiTFSI at *r* =1/6, while the optimal ionic conductivity of PEO/LiTFSI hybrids usually appears at *r* ~ 1/12 [13,28]. The higher ion conductivities of PVPS*_n_-b-*PEO_210_*-b-*PVPS*_n_*/LiTFSI than PEO/LiTFSI are different from other BCP electrolytes composed of PEO and a mechanically reinforced block like PS, in which the introduction of the reinforced phase usually impairs the ionic conductivity [13].

**Figure 5:**
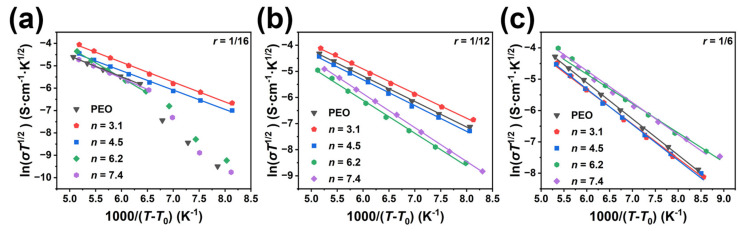
VTF plots for (**a**) PVPS−*b*−PEO−*b*−PVPS/LiTFSI with *r* = 1/16, (**b**) PVPS−*b*−PEO−*b*−PVPS/ LiTFSI with *r* = 1/12, and (**c**) PVPS−*b*−PEO−*b*−PVPS/LiTFSI with *r* = 1/6.

The Vogel–Tammann–Fulcher (VTF) equation was frequently utilized to describe the temperature dependence of the ionic conductivity of polymer electrolytes [66,67]. It can be written as follows:

(1) $$\ln(\sigma T^{1/2})=-\frac{E_{a}}{R}\frac{1}{T-T_{0}}+\ln A$$

where *T*_0_ represents the reference temperature set as *T*_g_—50 ℃, *E*_a_ is the apparent activation energy for ion transport, *A* is a pre-factor, and *R* is the gas constant, which equals 8.314 J/(mol·K). When ln(*σT*^1/2^) is plotted versus 1/(*T*-*T*_0_), a linear relationship is acquired. The slope of the line corresponds to −*E*_a_/*R*, and the intercept corresponds to ln*A*. The plots of logarithmic ionic conductivity against 1/(*T–T*_0_) for the hybrids are shown in Figure 5, and the calculated *E*_a_s are listed in Table S4. The obtained *E*_a_s in this work are comparable with the values (~10 kJ/mol) reported for other PEO-based SPEs [68,69]. We notice that at low PVPS fractions, *E*_a_s increases as the doping ratio increases, while for PVPS_6.2_*-b-*PEO_210_*-b-*PVPS_6.2_/LiTFSI and PVPS_7.4_*-b-*PEO_210_*-b-*PVPS_7.4_/LiTFSI, *E*_a_s follows different trends against *r*. It is counter-intuitive that *E*_a_s decreases with increasing doping ratio after *r* reaches 1/12, since restriction of chain segment motion resulting from superfluous lithium salt can be verified by increasing *T*_g_s (Table S2). This abnormal phenomenon is attributed to different salt distribution situations. For hybrids with low PVPS contents, lithium salt is mainly located in the PEO-rich phase, but at high PVPS fractions, PVPS blocks help with the dissociation of LiTFSI, thus decreasing *E*_a_s.

Table S5 summarizes the ionic conductivity and storage modulus of PVPS*-b-*PEO*-b-*PVPS/LiTFSI hybrids and other BCP-based SPEs reported in the literature. As we can see, the PVPS*-b-*PEO*-b-*PVPS/LiTFSI hybrids in this work exhibit a better balance in ionic conductivity and moduli as compared with most other BCP electrolyte materials. This shows that the integration of PVPS offers valuable insights for the development of novel SPEs for LIBs.

## 4. Conclusions

In summary, the solid electrolytes of LiTFSI-doped PVPS*-b-*PEO*-b-*PVPS ABA tri-BCP thermoplastic elastomers were successfully prepared. Asymmetric lamellar microphase-separated structures were formed in all the PVPS*-b-*PEO*-b-*PVPS tri-BCPs studied. By increasing the *f*_PVPS_ in the tri-BCPs, the lamellar structure becomes more ordered, while salt-doping weakens the tendency toward microphase separation. The melting temperature and glass transition temperature of the hybrids were influenced by the *f*_PVPS_, the spatial confinement of the microphase-separated structure, and the doping ratio. Due to the physical crosslinking domains formed by the glassy PVPS blocks, the moduli of the PVPS*-b-*PEO*-b-*PVPS/LiTFSI hybrids are greatly improved as compared with the PEO/LiTFSI hybrids. Moreover, the PVPS*-b-*PEO*-b-*PVPS/LiTFSI hybrids can outperform the PEO/LiTFSI hybrids in ionic conductivity due to the assistance of the PVPS blocks in the dissociation of Li^+^ ions. As a result, simultaneous improvement of mechanical properties and ionic conductivity can be achieved by the introduction of PVPS at both ends of PEO, which makes the PVPS*-b-*PEO*-b-*PVPS/LiTFSI hybrids have potential application in LIBs as solid electrolytes.

## Figures and Tables

**Table 1:** Details for PVPS*-b-*PEO*-b-*PVPS tri-BCPs.

Sample	Block *N* ^a^		*M*_n_^b^ (kg·mol^−1^)	*Ð* ^c^	*f* (%) of PVPS ^d^
	PVPS	PEO			
PVPS_3.1_*-b-*PEO_210_*-b-*PVPS_3.1_	3.1	210	11,400	1.02	12.9
PVPS_4.5_*-b-*PEO_210_*-b-*PVPS_4.5_	4.5	210	12,000	1.02	17.5
PVPS_6.2_*-b-*PEO_210_*-b-*PVPS_6.2_	6.2	210	12,800	1.02	23.0
PVPS_7.4_*-b-*PEO_210_*-b-*PVPS_7.4_	7.4	210	13,300	1.02	26.1

^a^ The number of repeating units was determined by ^1^H-NMR of P4VP*-b-*PEO*-b-*P4VP. ^b^ The number average molecular weight is calculated by ^1^H-NMR spectra. ^c^ Polydispersity of P4VP*-b-*PEO*-b-*P4VP, *Ð* = *M*_w_/*M*_n_ obtained from GPC. ^d^ The volume fraction of PVPS is calculated on the basis of the densities of PEO (1.128 g/cm^3^) and PVPS (1.16 g/cm^3^) [48,52].

## Data Availability

Data are contained within the article.

## Supplementary Materials

The following supporting information can be downloaded at: https://www.mdpi.com/article/10.3390/ma17092145/s1, Figures S1 and S2: ^1^H NMR spectra of TTCA and CTA-PEO-CTA and P4VP_6.2_*-b-*PEO*-b-*P4VP_6.2_; Figure S3: GPC traces of PEO-CTA_2_ and P4VP*-b-*PEO*-b-*P4VP tri-BCPs; Figure S4: FTIR spectra of PVPS_4.5_*-b-*PEO_210_*-b-*PVPS_4.5_ tri-BCP and PVPS_4.5_*-b-*PEO_210_*-b-*PVPS_4.5_/LiTFSI hybrids; Figure S5: Temperature-variable SAXS profiles of PVPS*_n_-b-*PEO_210_*-b-*PVPS*_n_*/LiTFSI hybrids with *r* = 1/16; Figure S6: Fitting result for PVPS_3.1_*-b-*PEO_210_*-b-*PVPS_3.1_/LiTFSI hybrid with *r* = 1/16 at 30 °C; Figure S7: Temperature-variable SAXS profiles of (a) PVPS_4.5_*-b-*PEO_210_*-b-*PVPS_4.5_/LiTFSI with *r* = 1/12 and (b) PVPS_4.5_*-b-*PEO_210_*-b-*PVPS_4.5_/LiTFSI with *r* = 1/6; Figure S8: Ionic conductivities of PVPS_5.8_/LiTFSI blends at different doping ratios *r* =0.1 and *r* = 0.9; Figure S9: Storage moduli of PEO_210_/LiTFSI hybrids, PEO_210_/PVPS_5.8_/LiTFSI blends and PVPS_6.2_*-b-*PEO_210_*-b-*PVPS_6.2_/LiTFSI at *r* = 1/6; Table S1: Detailed information for LiTFSI-doped tri-BCPs. Table S2: Thermal properties of salt-doped PEO and tri-BCPs collected by DSC; Table S3: Microphase separation morphology and grain size for PEO/LiTFSI and PVPS*-b-*PEO*-b-*PVPS/LiTFSI hybrids; Table S4: Apparent activation energy (*E*_a_) for ion transport for PEO/LiTFSI and PVPS*-b-*PEO*-b-*PVPS/LiTFSI hybrids; Table S5: Ionic conductivity and storage modulus compared to other block copolymer electrolytes around room temperature.
