# Supplementary material for: Poly(ethylene oxide)- and Polyzwitterion-Based Thermoplastic Elastomers for Solid Electrolytes
Source: Materials (Basel). 2024 May 3;17(9):2145. doi: 10.3390/ma17092145 (PMC11085580; doi:10.3390/ma17092145)
Supplement: Supplementary file 1 [file materials-17-02145-s001.zip › materials-2948742-supplementary.pdf]

Supplementary Materials

PEO- and Polyzwitterion-Based Thermoplastic Elastomers for Solid Electrolytes

Ding-Li Xia, Shi-Peng Ding, Ze Ye, Chen Yang and Jun-Ting Xu*

National Key Laboratory of Biobased Transportation Fuel Technology, International Research Center for X Polymers, Department of Polymer Science and Engineering, Zhejiang University, Hangzhou 310058, China

Table S1. Detailed information for LiTFSI-doped tri-BCPs.

Sample	r^a	$M_n{}^b$ (kg·mol^{-1})	$Đ^c$	f (%) of PVPS ^{d}
	1/16			9.9
$PVPS_{3.1}$-*b*-PEO_{210}-*b*-$PVPS_{3.1}$/LiTFSI	1/12	11400	1.02	9.1
	1/6			7.1
	1/16			13.5
$PVPS_{4.5}$-*b*-PEO_{210}-*b*-$PVPS_{4.5}$/LiTFSI	1/12	12000	1.02	12.5
	1/6			9.8
	1/16			17.9
$PVPS_{6.2}$-*b*-PEO_{210}-*b*-$PVPS_{6.2}$/LiTFSI	1/12	12800	1.02	16.7
	1/6			13.1
	1/16			20.5
$PVPS_{7.4}$-*b*-PEO_{210}-*b*-$PVPS_{7.4}$/LiTFSI	1/12	13300	1.02	19.2
	1/6			15.1

^{a} Doping ratio (r) defined according to r = [Li^+]/([EO]+[VPS]). ^{b} Number average molecular weight of P4VP-*b*-PEO-*b*-P4VP attained by ^{1}H-NMR spectra. ^{c} Polydispersity of P4VP-*b*-PEO-*b*-P4VP, $Đ = M_w/M_n$ obtained from GPC. ^{d} Volume fraction of PVPS calculated on the basis of the densities of PEO (1.128 g/cm^3), PVPS (1.16 g/cm^3) and LiTFSI (1.334 g/cm^3)[1, 2], we suppose that lithium salt prefers to enter and complex with the PEO phase.

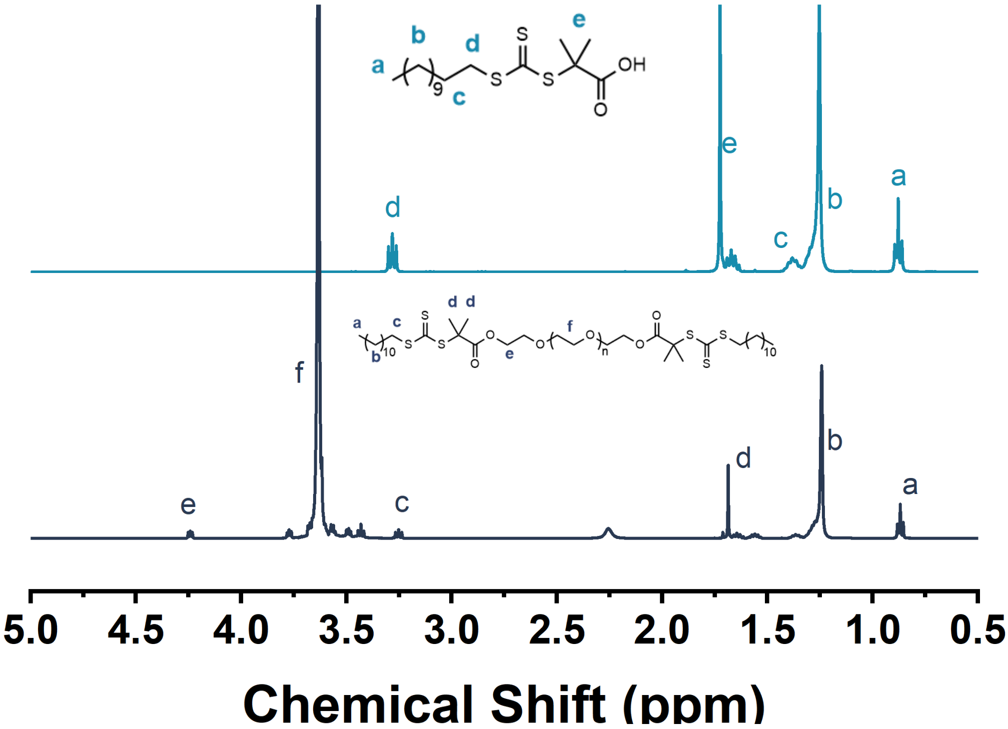

Figure S1. ^{1}H NMR spectra of TTCA and CTA-PEO-CTA in $CDCl_3$.

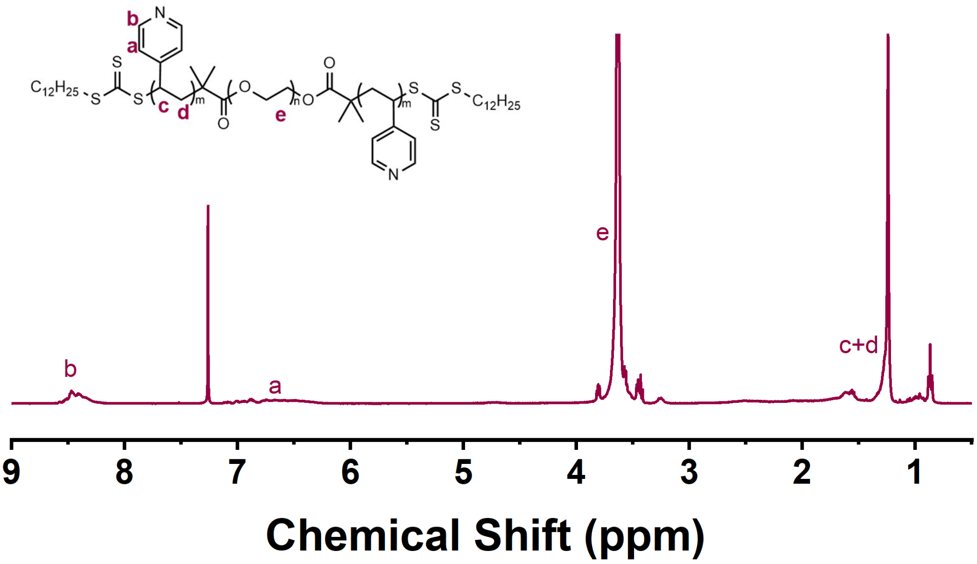

Figure S2. ^{1}H NMR spectrum of P4VP$_{6.2}$-*b*-PEO-*b*-P4VP$_{6.2}$ in $CDCl_3$.

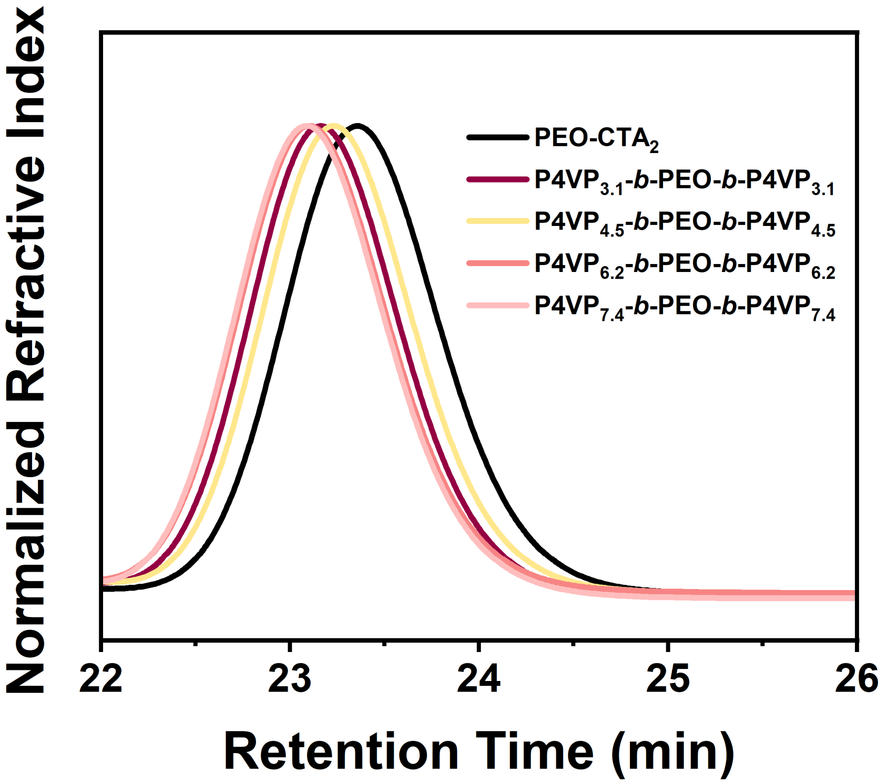

Figure S3. GPC traces of CTA-PEO-CTA and P4VP-*b*-PEO-*b*-P4VP tri-BCPs in DMF.

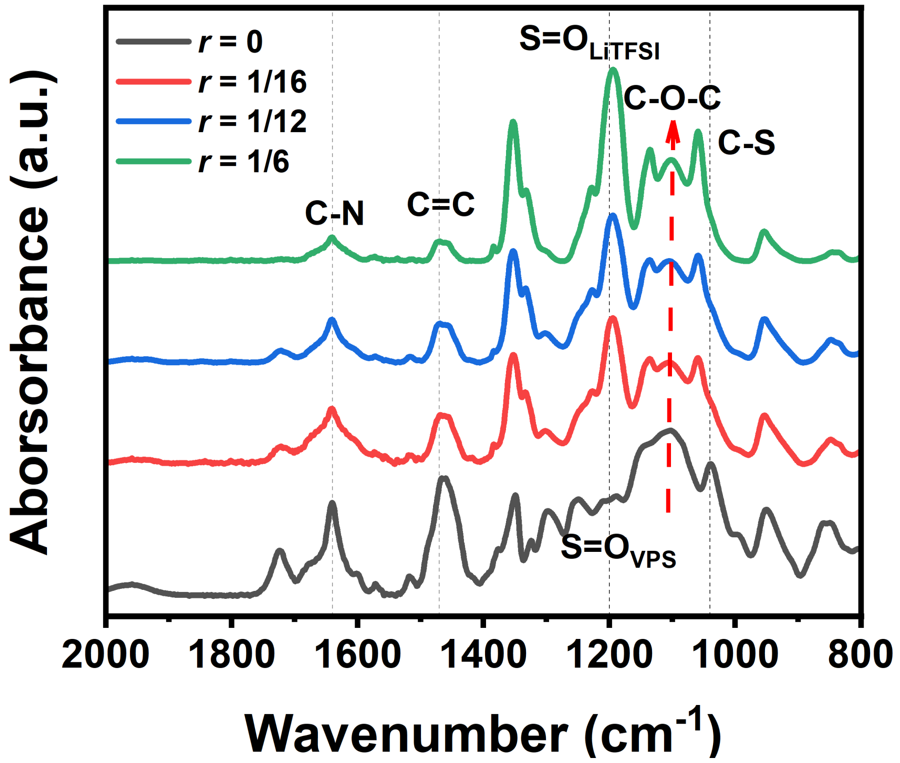

Figure S4. FTIR spectra of $PVPS_{4.5}$-*b*-PEO_{210}-*b*-$PVPS_{4.5}$ tri-BCP and $PVPS_{4.5}$-*b*-PEO_{210}-*b*-$PVPS_{4.5}$/LiTFSI hybrids.

We can see from **Figure S4** that, with the increase of salt content, there is no obvious shift of the absorption of C=N bond at 1630 cm^{-1} and C=C bond at 1470 cm^{-1}. Correspondingly, the shift of the absorption of C-O-C bond from 1110 cm^{-1} to 1100 cm^{-1} confirms the preferential complexion of Li^+ ion with C-O-C group rather than zwitterionic groups as doping ratio increases.

Table S2. Thermal properties of salt-doped PEO and tri-BCPs collected by DSC.

Sample	Doping Ratio (r)	Volume Fraction of PVPS	$T_{g, PEO}$ (°C)	$T_{c, PEO}$ (°C)	$T_{m, 1, PEO}$ (°C)	$\Delta H_{m, PEO}$ (J/g)	$X_{c, PEO}$ (%)
PEO	r = 1/16	-	-47.3	16.7	40.8	54.2	37.8
	r = 1/12	-	-44.0	-6.8	-4.1	7.1	5.4
	r = 1/6	-	-38.5	-23.7	-17.4	2.3	2.4
$PVPS_{3.1}$-*b*-PEO_{210}-*b*-$PVPS_{3.1}$	r = 1/16	0.099	-42.9	8.6	32.1	26.7	21.7
	r = 1/12	0.091	-43.0	-6.1	-2.3	3.4	3.0
	r = 1/6	0.071	-36.9	-18.4	-12.3	2.3	2.7
$PVPS_{4.5}$-*b*-PEO_{210}-*b*-$PVPS_{4.5}$	r = 1/16	0.135	-42.9	10.7	31.6	23.7	20.1
	r = 1/12	0.125	-44.1	-6.1	-1.5	7.5	6.9
	r = 1/6	0.098	-37.6	-15.9	-13.2	2.2	2.6
$PVPS_{6.2}$-*b*-PEO_{210}-*b*-$PVPS_{6.2}$	r = 1/16	0.179	-44.5	16.7	35.9	33.1	29.6
	r = 1/12	0.167	-45.2	-7.1	-2.1	5.3	5.1
	r = 1/6	0.131	-36.1	-19.7	-11.3	1.8	2.3
$PVPS_{7.4}$-*b*-PEO_{210}-*b*-$PVPS_{7.4}$	r = 1/16	0.205	-43.2	18.7	35.3	31.8	29.4
	r = 1/12	0.192	-40.4	9.0	30.4	20.2	20.1
	r = 1/6	0.151	-32.2	-17.4	-8.2	1.9	2.4

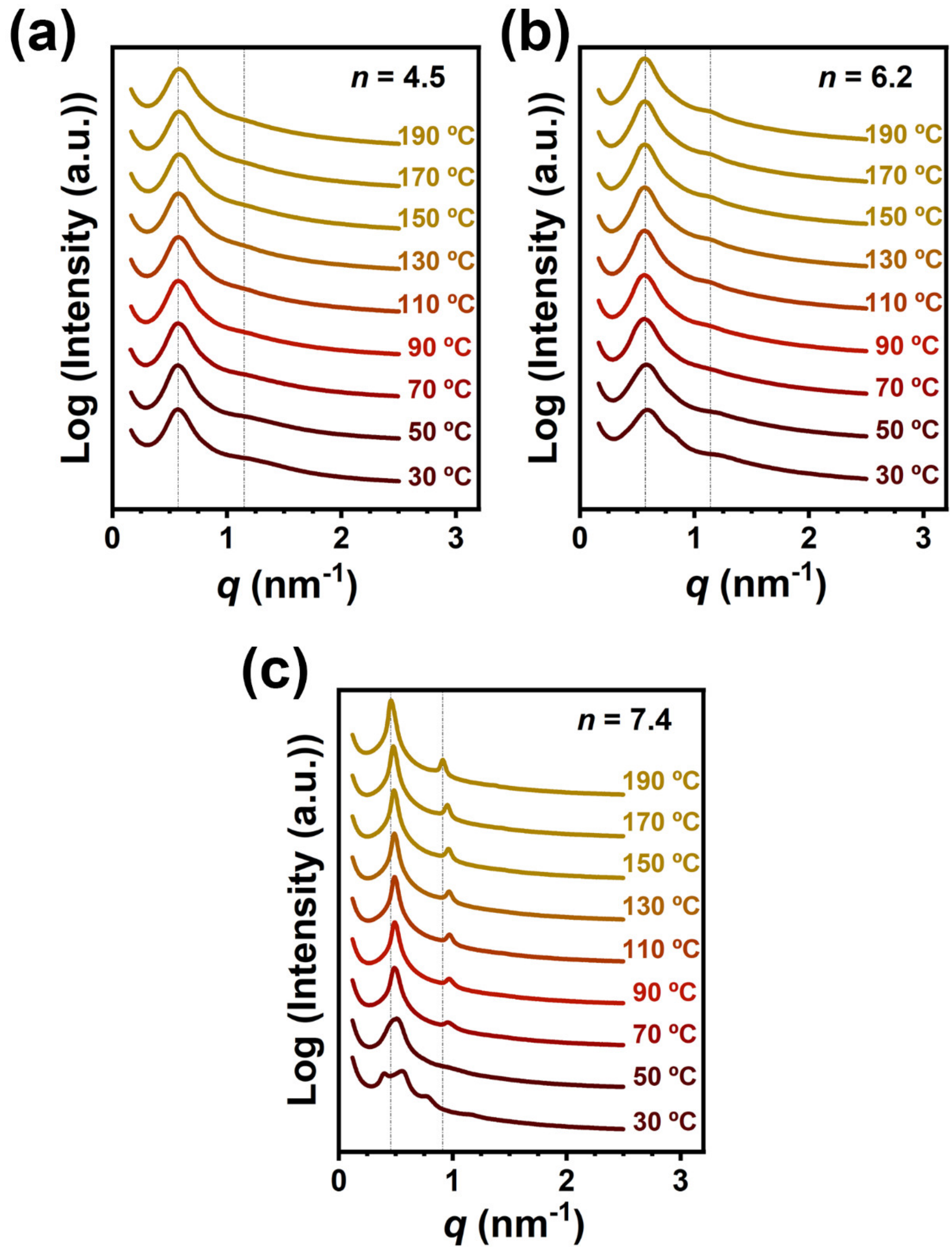

Figure S5. Temperature-variable SAXS profiles of $PVPS_n$-*b*-PEO_{210}-*b*-$PVPS_n$/LiTFSI hybrids with r = 1/16 from 30 °C to 200 °C. (a) n = 4.5, (b) n = 6.2, (c) n = 7.4.

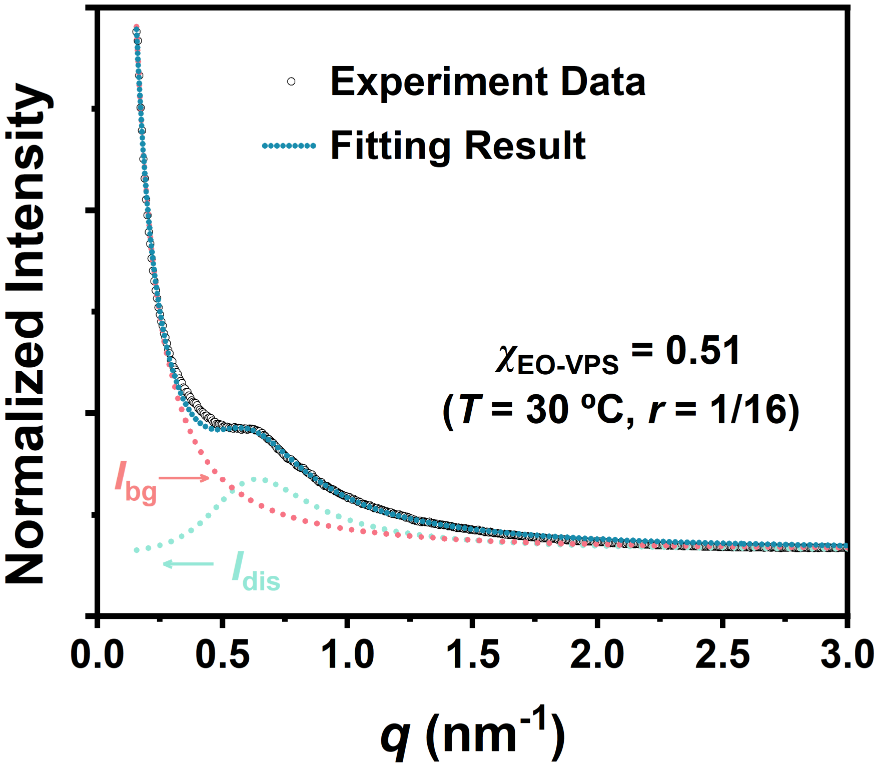

Figure S6. Fitting result for $PVPS_{3.1}$-*b*-PEO_{210}-*b*-$PVPS_{3.1}$/LiTFSI hybrid with r = 1/16 at 30 °C.

According to Ref. 5, for PS-*b*-PEO/LiTFSI hybrids with molecular weight around 10 kg mol^{-1}, at doping ratio of 1/16 and 30 °C, the increase in χ_{eff} with r can be described by following equation

$$\chi_{eff} = A(T) + \frac{B(T)}{N} + \frac{C(T)}{N}\left[1 - \exp\left(\frac{-D(T)r}{N}\right)\right] \qquad \text{(S1)}$$

where $A(T) = 10.2 \times T^{-1}$, $B(T) = 1.85 \times 10^3 \times T^{-1}$, $C(T) = 1.01 \times 10^{-2} \times T$ and $D(T) = 22.4 \times T$. M_{PS} and M_{PEO} are set as 4.9 and 5.5 kg mol^{-1}, respectively, so that N ~~is~~ equals ~~to~~ 96.[3-5]

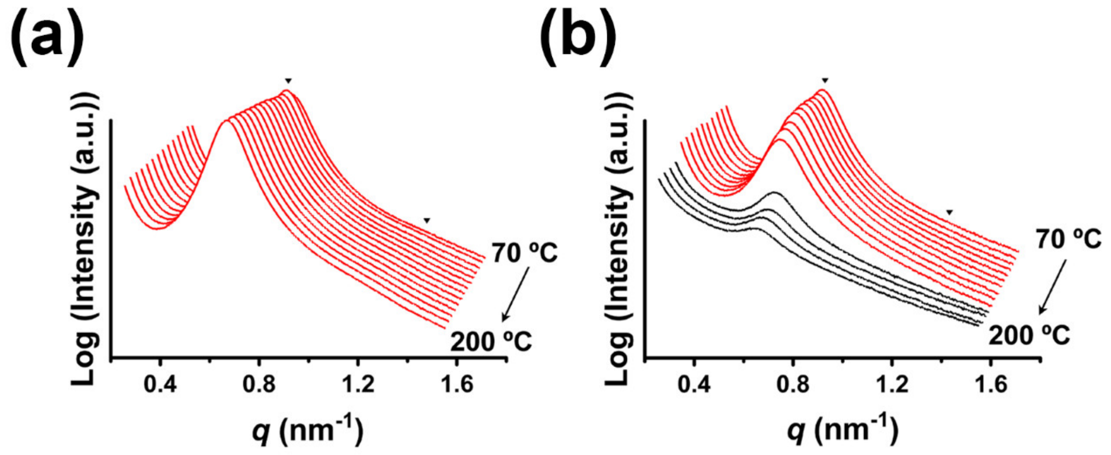

Figure S7. Temperature-variable SAXS profiles of (a) $PVPS_{4.5}$-*b*-PEO_{210}-*b*-$PVPS_{4.5}$/LiTFSI with r = 1/12 and (b) $PVPS_{4.5}$-*b*-PEO_{210}-*b*-$PVPS_{4.5}$/LiTFSI with r = 1/6.

Table S3. Microphase separation morphology and grain size for PEO/LiTFSI and PVPS-*b*-PEO-*b*-PVPS/LiTFSI hybrids.

Sample	*r*	Ionic Conductivity (S/cm) at 30°C	Grain Size (nm)	Morphology
**PEO_{210}/LiTFSI**	**1/16**	4.35×10^{-5}	-	-
	**1/12**	4.63×10^{-5}	-	-
	**1/6**	2.16×10^{-5}	-	-
**$PVPS_{3.1}$-PEO_{210}-$PVPS_{3.1}$/LiTFSI**	**1/16**	7.33×10^{-5}	9.7	dis
	**1/12**	6.17×10^{-5}	-	homo
	**1/6**	1.77×10^{-5}	-	homo
**$PVPS_{4.5}$-PEO_{210}-$PVPS_{4.5}$/LiTFSI**	**1/16**	5.30×10^{-5}	32.0	LAM
	**1/12**	4.03×10^{-5}	29.8	LAM
	**1/6**	1.94×10^{-5}	31.4	LAM → dis
**$PVPS_{6.2}$-PEO_{210}-$PVPS_{6.2}$/LiTFSI**	**1/16**	6.08×10^{-6}	27.1	LAM
	**1/12**	1.09×10^{-5}	38.5	LAM
	**1/6**	3.97×10^{-5}	34.0	LAM
**$PVPS_{7.4}$-PEO_{210}-$PVPS_{7.4}$/LiTFSI**	**1/16**	3.53×10^{-6}	23.1	LAM
	**1/12**	8.79×10^{-6}	74.6	LAM
	**1/6**	3.29×10^{-5}	68.6	LAM

Grain size (L) can be calculated by Scherrer equation[6, 7]

$$L = 2\pi K(\mathrm{FWHM})^{-1} \qquad \text{(S2)}$$

where K is a constant taking the value of 0.93. FWHM represents the half-peak width of the primary scattering peak in the SAXS patterns.

Table S4. Apparent activation energy (E_a) for ion transport for PEO/LiTFSI and PVPS-*b*-PEO-*b*-PVPS/LiTFSI hybrids.

Sample	Doping ratio (*r*)	E_a (kJ mol^{-1})
	1/16	7.7
PEO_{210}/LiTFSI	1/12	8.2
	1/6	9.6
	1/16	7.4
$PVPS_{3.1}$-PEO_{210}-$PVPS_{3.1}$/LiTFSI	1/12	7.8
	1/6	9.5
	1/16	7.2
$PVPS_{4.5}$-PEO_{210}-$PVPS_{4.5}$/LiTFSI	1/12	8.2
	1/6	9.2
	1/16	11.2
$PVPS_{6.2}$-PEO_{210}-$PVPS_{6.2}$/LiTFSI	1/12	10.5
	1/6	8.5
	1/16	8.4
$PVPS_{7.4}$-PEO_{210}-$PVPS_{7.4}$/LiTFSI	1/12	10.8
	1/6	7.8

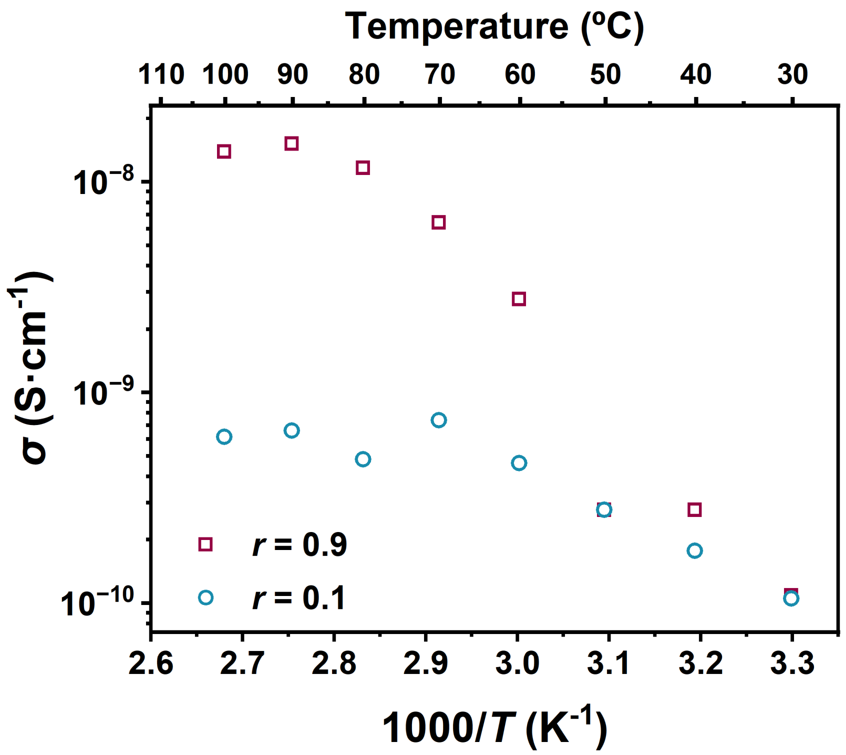

Figure S8. Ionic conductivities of $PVPS_{5.8}$/LiTFSI blends at different doping ratios, r =0.1 and r = 0.9.

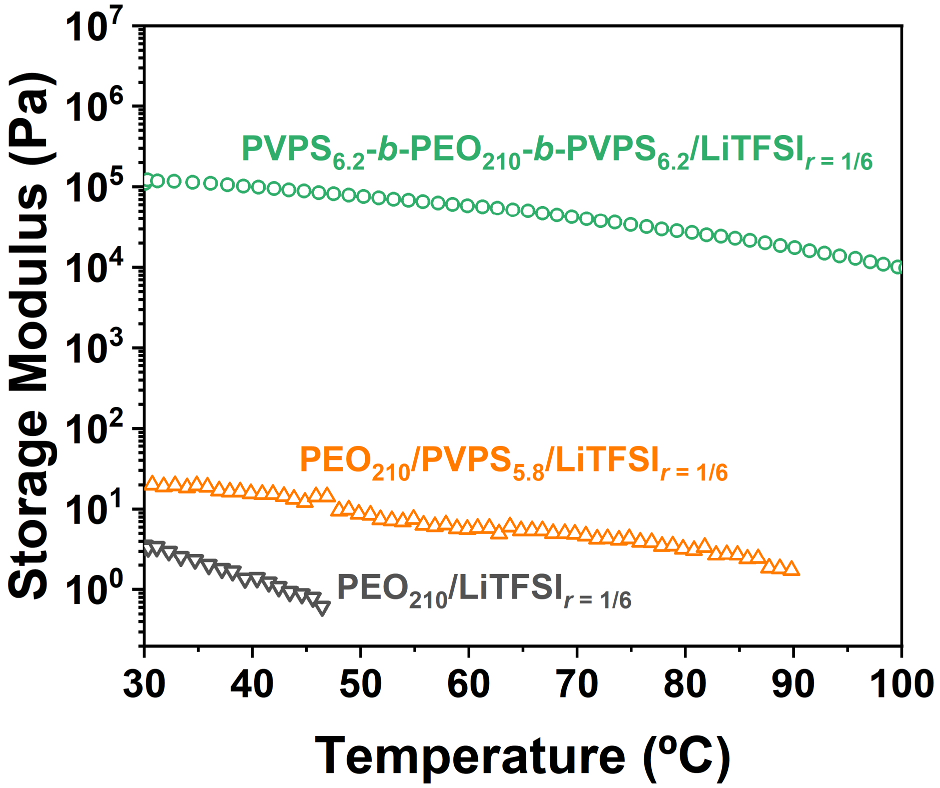

Figure S9. Storage moduli of PEO_{210}/LiTFSI hybrids, PEO_{210}/$PVPS_{5.8}$/LiTFSI blends and $PVPS_{6.2}$-*b*-PEO_{210}-*b*-$PVPS_{6.2}$/LiTFSI at $r = 1/6$.

Employing a methodology akin to that used for PEO/LiTFSI electrolytes, we formulated PEO/PVPS/LiTFSI electrolyte. We incorporated LiTFSI in a quantitative manner, adhering to the molar ratio of LiTFSI to the total repeating units of PEO and PVPS. The weight fraction of PVPS in the PEO/PVPS/LiTFSI hybrid was 0.124, consistent with that in the tri-BCP counterpart. We subsequently assessed the rheological properties of these composites, the results of which are delineated subsequently in **Figure S9**.

The robust intra- and intermolecular interactions confer PVPS a glassy state at ambient temperature, thereby establishing it as a physical crosslinking site within the blend. Consequently, despite the blend retaining a viscoelastic liquid-like character, the modulus experiences an approximate tenfold increase against PEO/LiTFSI electrolyte. However, the mechanical reinforcement imparted by the PVPS in the blend is still significantly inferior to that achieved by PVPS in the corresponding tri-

BCP as PEO and PVPS are chemically bonded to each other in the tri-BCP, leading to the formation of lamellar structures as a result of microphase separation.

Table S5. Ionic conductivity and storage modulus compared to other block copolymer electrolytes around room temperature.[8-14]

Electrolyte[a]	σ (S/cm)	G' (Pa)	Ref.
$PVPS_{3.1}$-*b*-PEO_{210}-*b*-$PVPS_{3.1}$/LiTFSI	7.3×10^{-5} (30 °C)	2.9×10^{3} (30 °C)	-
$PVPS_{6.2}$-*b*-PEO_{210}-*b*-$PVPS_{6.2}$/LiTFSI	3.9×10^{-5} (30 °C)	1.1×10^{5} (30 °C)	-
PEO/LiTFSI	~1×10^{-7} (30 °C)	1×10^{6} (30 °C)	8
PS-*b*-PEO/LiTFSI	1.2×10^{-5} (30 °C)	1×10^{7} (30 °C)	9, 10
PS-*b*-POEGMA/$LiClO_4$	1×10^{-5} (30 °C)	2×10^{4} (25 °C)	11
gPS-*b*-gPEO-*b*-gPS/LiTFSI	2×10^{-5} (25 °C)	3×10^{4} (45 °C)	12
PPMTC-*b*-PEO/LiTFSI	2×10^{-4} (30 °C)	3×10^{1} (30 °C)	13
PC-*b*-PEO-*b*-PC[b]/LiTFSI	6.7×10^{-4} (30 °C)	5.2×10^{5} (30 °C)	14

[a] Salt content varies; parameters reported for electrolytes at their optimized salt ratio for ionic conductivity. [b] PC represents a kind of CO_2-derived polycarbonate, poly(4-vinyl cyclohexene oxide carbonate).

Reference

1. Timachova, K.; Villaluenga, I.; Cirrincione, L.; Gobet, M.; Bhattacharya, R.; Jiang, X.; Newman, J.; Madsen, L.A.; Greenbaum, S.G.; Balsara, N.P. Anisotropic Ion Diffusion and Electrochemically Driven Transport in Nanostructured Block Copolymer Electrolytes. *J. Phys. Chem. B* **2018,** *122*, 1537-1544.
2. Ding, S.P.; Zhang, Z.K.; Ye, Z.; Du, B.Y.; Xu, J.T. Fabrication of High χ-Low N Block Copolymers with Thermally Stable Sub-5 nm Microdomains Using Polyzwitterion as a Constituent Block. *ACS Macro Lett.* **2021,** *10*, 1321-1325.
3. Wang, R.Y.; Zhang, Z.K.; Guo, X.S.; Cao, X.H.; Zhang, T.Y.; Tong, Z.Z.; Xu, J.T.; Du, B.Y.; Fan, Z.Q. Mechanistic Study of the Influence of Salt Species on the Lower Disorder-to-Order Transition Behavior of Poly(Ethylene Oxide)-*b*-Poly(Ionic Liquid)/Salt Hybrids. *Macromolecules* **2020,** *53*, 4560-4567.
4. Chintapalli, M.; Timachova, K.; Olson, K.R.; Mecham, S.J.; Desimone, J.M.; Balsara, N.P. Lithium Salt Distribution and Thermodynamics in Electrolytes Based on Short Perfluoropolyether-*bloc*k-Poly(Ethylene Oxide) Copolymers. *Macromolecules* **2020,** *53*, 1142-1153.
5. Teran, A.A.; Balsara, N.P. Thermodynamics of Block Copolymers with and without Salt. *J. Phys. Chem. B* **2014,** *118*, 4-17.
6. Chintapalli, M.; Chen, X.C.; Thelen, J.L.; Teran, A.A.; Wang, X.; Garetz, B.A.; Balsara, N.P. Effect of Grain Size on the Ionic Conductivity of a Block Copolymer Electrolyte. *Macromolecules* **2014,** *47*, 5424-5431.
7. Grundy, L.S.; Fu, S.; Galluzzo, M.D.; Balsara, N.P. The Effect of Annealing on the Grain Structure and Ionic Conductivity of Block Copolymer Electrolytes. *Macromolecules* **2022,** *55*, 10294-10301.
8. Pesko, D.M.; Webb, M.A.; Jung, Y.K.; Zheng, Q.; Miller, T.F., Iii; Coates, G.W.; Balsara, N.P. Universal Relationship between Conductivity and Solvation-Site Connectivity in Ether-Based Polymer Electrolytes. *Macromolecules* **2016,** *49*, 5244-5255.
9. Bouchet, R.; Phan, T.N.T.; Beaudoin, E.; Devaux, D.; Davidson, P.; Bertin, D.; Denoyel, R. Charge Transport in Nanostructured PS-PEO-PS Triblock Copolymer Electrolytes. *Macromolecules* **2014,** *47*, 2659-2665.
10. Singh, M.; Odusanya, O.; Wilmes, G.M.; Eitouni, H.B.; Gomez, E.D.; Patel, A.J.; Chen, V.L.; Park, M.J.; Fragouli, P.; Iatrou, H.; Hadjichristidis, N.; Cookson, D.; Balsara, N.P. Effect of Molecular Weight on the Mechanical and Electrical Properties of Block Copolymer Electrolytes. *Macromolecules* **2007,** *40*, 4578-4585.
11. Rolland, J.; Brassinne, J.; Bourgeois, J.P.; Poggi, E.; Vlad, A.; Gohy, J.F. Chemically Anchored Liquid-Peo Based Block Copolymer Electrolytes for Solid-State Lithium-Ion Batteries. *J. Mater. Chem. A* **2014,** *2*, 11839-11846.
12. Bates, C.M.; Chang, A.B.; Momcilovic, N.; Jones, S.C.; Grubbs, R.H. Aba Triblock Brush Polymers: Synthesis, Self-Assembly, Conductivity, and Rheological Properties. *Macromolecules* **2015,** *48*, 4967-4973.
13. Cao, X.H.; Li, J.H.; Yang, M.J.; Yang, J.L.; Wang, R.Y.; Zhang, X.H.; Xu, J.T. Simultaneous Improvement of Ionic Conductivity and Mechanical Strength in

Block Copolymer Electrolytes with Double Conductive Nanophases. *Macromol. Rapid Commun.* **2020,** *41*, 1900622.

14. Gregory, G.L.; Gao, H.; Liu, B.; Gao, X.; Rees, G.J.; Pasta, M.; Bruce, P.G.; Williams, C.K. Buffering Volume Change in Solid-State Battery Composite Cathodes with CO_2-Derived Block Polycarbonate Ethers. *J. Am. Chem. Soc.* **2022,** *144*, 17477-17486.
